# Consolidation of LVFRT capabilities of microgrids using energy storage devices

**DOI:** 10.1038/s41598-023-49659-0

**Published:** 2023-12-15

**Authors:** Aya M. Moheb, Enas A. El-Hay, Attia A. El-Fergany

**Affiliations:** Electrical Power and Machines Department, Faculty of Engineering, Zagazig University, Zagazig, 44519 Egypt

**Keywords:** Engineering, Mathematics and computing

## Abstract

The performance and dependability of distribution networks may be enhanced by the incorporation of microgrids (MGs). However, it is necessary to enhance low voltage fault-ride-through (LVFRT), which has the capacity to prevent abrupt grid disconnections during LV occurrences under problematic circumstances. In this study, a control strategy for energy storage elements (ESDs) which includes batteries and supercapacitors is proposed to enhance LVFRT under balanced and unbalanced faults. The MG comprises wind farms and/or photovoltaic arrays. Based on the dynamic simulations using MATLAB/SIMULINK, the ESDs can enhance LVFRT capability. A comparison of the conventional crowbar scheme and ESDs is realized, and the latter has a better performance than the former in retaining the DC-link voltage within satisfactory bounds. For the purpose of maintaining the DC-link voltage at a reference level, the battery stores extra power in the DC-bus of three systems. LVFRT is improved by the crowbar circuit, however the resistance consumes the extra power. Super capacitors (SCs) prevent DC voltage fluctuations, reduce active power oscillations, and hasten system stabilization when present. At an advanced stage of this effort, the coot bird optimizer (CBO) is applied to generate the best gains of bi-directional converter PI-controller and the ESDs ratings to have minimum ripples in the DC-bus voltage and to boost the LVFRT capability of the MGs. The viability of the proposed method based on the CBO’s results is indicated with further validations under different operating scenarios.

## Introduction

The significance of electric grid (egrid) codes is that they run the egrid in line with the utmost stability and to adjust frequency and terminal voltage. Low voltage fault ride-through (LVFRT) ability is among the most required egrid codes. This prevents loss in generated electricity and helps generation units connect to the egrid in a particular way during and after a fault occurs^1^.

Photovoltaic (PV) generating arrays and double-fed induction generator (DFIG) based wind systems have a special concern for renewable energy sources in the world as they serve to diminish the impacts of greenhouse gas emissions^2^.

Using a PV involves converting the irradiation of sunlight through one or two layers of semiconductor materials. A two-stage PV system includes a DC/DC converter and a DC/AC inverter to generate electrical power^3^. Tracking PV arrays for maximum power (MP) is accomplished with MPPT procedures. These are the perturb-and-observe technique^4^, the constant voltage technique^5^, and the incremental conductance technique^6^.

Several approaches are introduced to boost the LVFRT capability of DFIG, especially since it is very sensitive to egrid faults^7^. LVFRT strategies are divided into software and hardware methods. Software methods limit inrush rotor current, which are the feedforward control technique^8^, vector technique^9^, sliding control technique^10^, fuzzy logic^11^, and the linear quadratic regulator^12^. Hardware devices such as protective, and reactive power devices, and energy storage elements (ESDs) are presented. Protective devices limit rises in rotor currents, and DC voltage^13^, DC chopper resistance^14^, dynamic braking resistor^15^, superconducting current limiter^16^, and bridge fault current limiter^17^. The reactive power is injected into DFIG to enhance the performance through devices like static var compensation and static synchronous compensation (STATCOM)^18^, dynamic voltage resistance (DVR)^19^, and unified power quality conditioner^20^. During a fault, ESDs rely on storing active power that is stored by batteries^21^, supercapacitors (SC)^22^, flywheel energy storage (FES)^23^, superconducting magnetic energy storage (SMES)^3^ and compressed air energy storage^24^. FESs are efficient, less maintained, high response time and not affected by repeating charge and discharge. Its disadvantages are a high cost and a short life span. On the other hand, SC is an important source of reactive power when voltage level is decreased than its rated value. Its efficiency is around 85–95%. It is an important source of reactive power. It has long lifetime. It is not affected by charging and discharging rate. It has high power density compared by other types. It has less energy density^22^. Compressed air energy storage is higher power density. Its efficiency is 80–90%, but it has high capital cost^24^.

Approaches proposed for enhancing PV systems are based on improving control methods and adding external devices as well. Improved control methods include feedback linearization control^25^, fuzzy logic control^26^, adaptive DC-link voltage control strategy^27^ and modified inverter control^28^. External devices are added using ESDs such as batteries^21,29^ and SC^30^, injection reactive power devices like FACTS devices^31^, and protective devices (e.g. fault current limiters^31^, dynamic braking resistors^32^, and DC-link crowbar (CB) circuit^33^).

To adapt to the expansion of energy sources, microgrids (MGs) are utilized to boost the reliability and stability of power systems that operate in two-mode egrid-connecting mode or islanding mode. A MG includes wind farms and/or PV array systems, both of which have numerous advantages, including their high flexibility and scalability for different purposes, as well as power management capabilities^34^.

Numerous approaches are introduced to enhance the LVFRT capability of PV-wind MGs. As in^35^, a hybrid PV/WT based on a SC ESD is proposed to flatten power instabilities under varied wind speed and solar irradiance. SC has a quick response time for power regulation^36^. In^37^, improving the LVFRT capability of hybrid PV/wind using STATCOM is being explored. As in^38^, LVFRT capability of hybrid PV-wind with new control methods is proposed. However, LVFRT capability of hybrid PV-wind with adaptive DC-link control is proposed in^34^. As presented in^39^, hybrid wind-PV based on a lithium-ion battery is presented. As in^40^, the LVFRT capability of hybrid PV/WT using CB circuit is discussed. As reported in^41^, LVFRT capability of hybrid PV/WT using DVR is proposed.

Currently, DFIG controls 50% of all installed wind energy conversion systems, leading all other forms of WTG installations globally^42^. Because its stator is directly coupled to the power egrid and its rotor is linked to a back-to-back (BtB) converter, the DFIG is particularly vulnerable to egrid faults. The DFIG is principally sensitive to power egrid faults as its stator is straightforward coupled to the power egrid and its rotor is linked to BtB converter. This is the most used generator that has been applied to wind turbines that work at sub and super synchronous speeds, and its converter handles with 30% of its ratings so power losses are low^7^. DFIG is simple controllability of the active and reactive power, controllability of the grid and generator sides, and functionality under fluctuating wind speed^42,43^.

Batteries are superior device as they are capable of storing excess energy, and reduce the amplitude of AC current. The disadvantages of batteries are short life span if the battery is discharged deeply, fluctuation in DC parameters, and has a slow time response, so it cannot provide frequency support^44,45^. A lithium-ion battery is the most commonly used to enhance the dynamic performance of DFIG according to decision matrix (DM) criteria in^46^.

SMES is a DC control device that stores electrical energy into an electromagnetic field when the direct current has flowed in a superconductor coil. The merits of SMES are high Cyclic efficiency (90–95%), large power density, short response time, and unlimited charging and discharging cycle. It has a high capital cost. To ensure efficient application of SMES, suitable power system locations must be selected carefully in the power system^7^.

Various optimization algorithms are for the use of getting the optimal sizing of an off-grid with PV panels, wind turbines, and battery. In^47^, a hybrid genetic algorithm with particle swarm optimizer is applied to get the optimal size of hybrid PV panels, wind turbines, and battery. In^48^, genetic algorithm (GA) is applied to optimize the design variables of hybrid solar/wind/battery system (number of the PV modules, number of wind turbines, number of batteries, the PV module slope angle, and the wind turbine installation.

The coot bird optimizer (CBO) is an intelligent optimization algorithm which used to improve the LVFRT capability of the studied systems. The CBO is used to determine the suitable ratings of both the battery and the super capacitor according to decrease ripples in the DC-link voltage. Choosing the suitable ratings of both the battery and the super capacitor has a great effect on the DC-bus voltage.

This article presents a methodology for the performance improvement for LVFRT of PV, DFIG based WT, and hybrid PV/WT on egrid mode using CB protection, batteries, and SCs. MATLAB/Simulink is employed to imitate the performance of the three systems in different cases during symmetrical and asymmetrical failures. The dynamic model of each system is discussed. The CBO is implemented to decide the optimal size of battery, and SCs. Moreover, the CBO is applied to generate the best gains of bi-directional converter PI-controller and the ESDs ratings to have minimum ripples in the DC bus voltage and to boost the LVFRT capability of the MGs.

The text of this article is coordinated as follows: “System configuration and modelling” announces the system's construction and model. “Results and discussions” demonstrates the numerical results and discussions. “Procedures of the CBO with problem formulation” publicizes the procedures of CBO with problem formulation, and “Conclusions” concludes the final view plus future trend of this effort.

## System configuration and modelling

**Figure 1:**
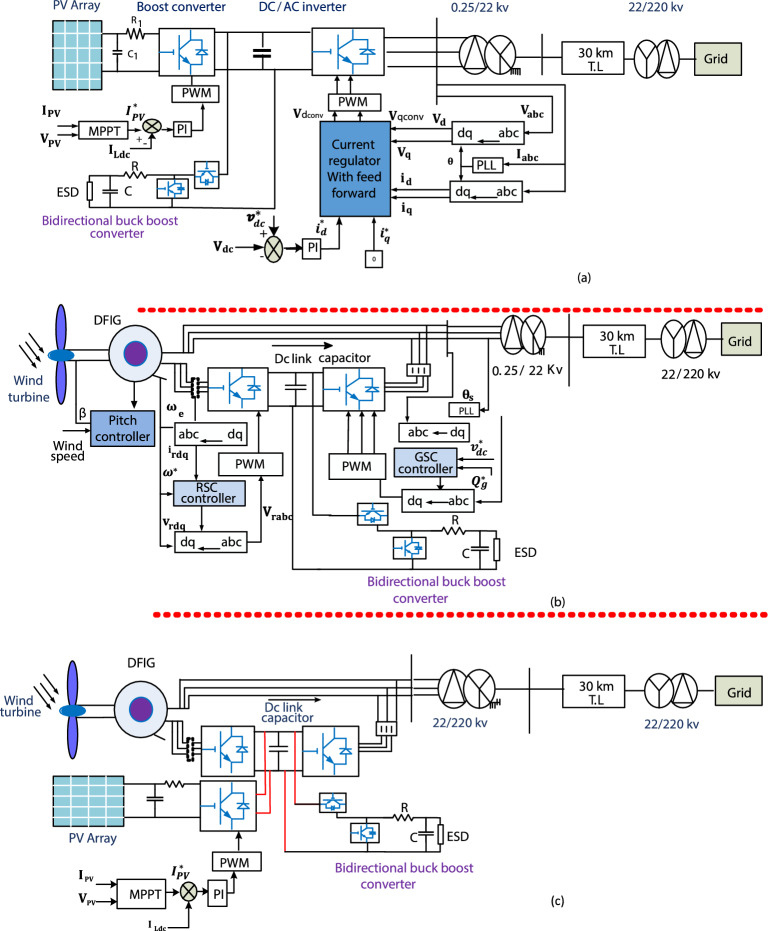
System description: (**a**) PV on-egrid mode, (**b**) DFIG based-WT on-egrid mode, and (**c**) hybrid PV/WT on-egrid mode.

Figure 1a–c shows three systems of a 100 kW PV on-egrid mode, a 6 MW DFIG-WT on-egrid mode, and a 1.4 MW hybrid PV/WT on egrid mode. They are detailed as follows.

PV system is comprised of a PV cell, a DC/DC boost converter, and a DC/AC inverter, all of which are associated to crop the MP at various sun radiation levels. The DC/DC converter can be regulated by an MPPT controller, which ensures MP saving in different environments. The DC/AC inverter dominates the DC bus voltage and controls the injected reactive power to the system by applying the voltage-oriented control (VOC) technique. The system is associated to the egrid through a 0.25/22 kV step-up transformer, and a 30 km transmission line^49^. The parameters of PV system are illustrated in Appendix (A1).

A 6 MW wind farm is presented in Fig. 1b. The DFIG-WT system is consisted of a wind turbine, a drive train system, a pitch controller system, a speed control system, DFIG, RSC, and GSC all of which are connected to control wind power and inject the produced power into the egrid at a constant frequency. The rotor converter captures the MP at different wind speeds, while the egrid converter regulates the substituted reactive power with the egrid. The parameters of wind farm are illustrated in Appendix (A1).

The hybrid PV/WT on-egrid mode consists of a 1 MW WT and a 400 kW PV system linked to the grid via 30 km of transmission lines and a 0.25/22 kV D/Y step-up transformer. As shown in Fig. 1c, the PV system is connected to WT via buck boost converter to 1150 DC V bus. The ESD keeps up with the dc-link voltage of the hybrid system via the bi-directional DC/DC converter to improve LVFRT ability.

### Modelling of DFIG-WT and its conversion system

The mathematical model of WT is derived by extracting the mechanical output power ^50,51^ as follows.

1 $${P}_{mec}=\frac{\rho }{2}\pi {R}^{2}{v}_{w}^{3}{c}_{p}(\lambda \cdot \beta )$$

2 $$\lambda =\frac{{R\omega }_{r}}{{v}_{w}}$$

3 $${C}_{p}\left(\lambda /\beta \right)=0.5176\left(\frac{116}{\lambda }+0.4\beta -5\right){e}^{\frac{-21}{\lambda }}+0.00668\lambda )$$

4 $$\frac{1}{\lambda }=\frac{1}{\lambda +0.08\beta }-\frac{0.035}{{\beta }^{3}+1}$$

**Figure 2:**
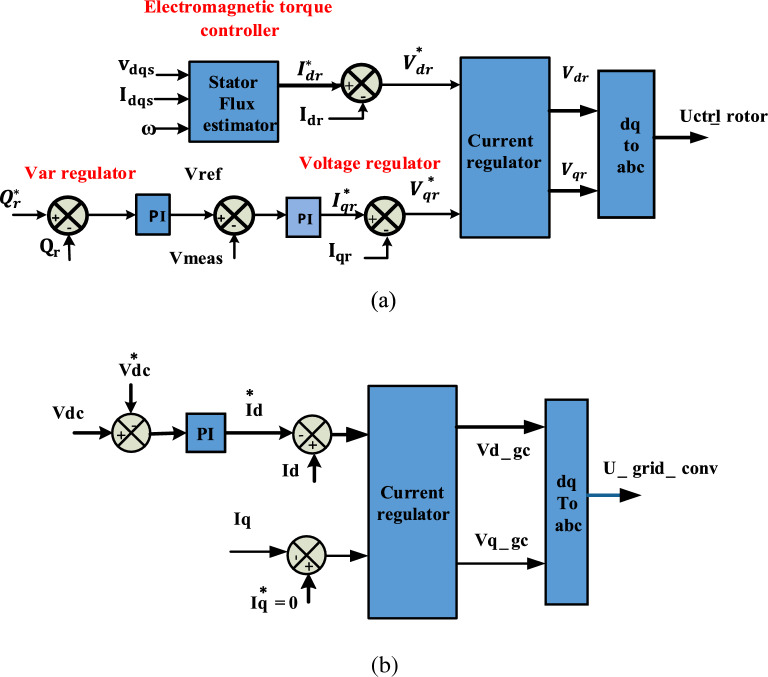
Control schemes of (**a**) rotor side converter, (**b**) grid side converter.

As DFIG provides variable speed operation, it can operate at sub-synchronous and super-synchronous speeds, so it is the most commonly used generator in wind systems. In DFIG, the stator is directlylinked to the egrid and its rotor is associated to a BtB converter, thus making it more economical. DFIG and its conversion system are in Fig. 1b. The mathematical model for DFIG according to d–q reference is in^46,52^. The DFIG BtB converter controls active and reactive power through vector technique to achieve a varying speed operation as shown in Fig. 1b. RSC controls electromagnetic torque in the q axis and converts stator reactive power through the d axis. Three-phase rotor current ${i}_{rabc}$ is transformed to rotor current in d–q axis (${i}_{qr}, {i}_{dr}$). The reference power (${p}_{s}^{*}$), which is derived from the MPPT curve, is compared with the measured value (${P}_{S}$) to get ${i}_{qr}^{*}$. The reactive power is controlled by comparing ${Q}_{s}^{*}$ with both ${Q}_{S}$ to get ${i}_{dr}^{*}\cdot {i}_{qr}^{*}$ and ${i}_{qr}$ are compared to get error signals which are sent to the PI-controller to get ${V}_{dr}$ and also the same for ${i}_{dr}^{*}$ and ${i}_{dr}$ to get ${V}_{qr}$. The (${V}_{dr}$, ${V}_{qr}$) signals are sent to PWM to obtain the RSC signal as revealed in Fig. 2a. The GSC adjusts DC voltage at its rated value and controls reactive power, which is substituted between the GSC and the egrid. The rated value of DC voltage (${v}_{dc}^{*}$) is compared with the measured value of dc voltage to get ${i}_{dg}^{*}$. ${Q}_{g}^{*}$ is compared with ${Q}_{g}$ to get ${i}_{g}^{*}$. ${I}_{qg}$ is compared to ${i}_{qg}^{*}$ to getting an error signal and sending it to the PI-controller to get ${v}_{qg}$. The same is true for ${I}_{dg}$ and ${i}_{dg}^{*}$ to get ${v}_{dg}$. Current regulators adjust error signals (${v}_{qg},{v}_{qg}$ ) and send them to PWM to get GSC switch signal as publicized in Fig. 2b.

The stator and rotor voltage equations in the d–q reference are as follows.

5 $${v}_{sd}={R}_{s}{i}_{sd}-{\omega }_{s}{\lambda }_{sq}+\frac{d{\lambda }_{sd}}{dt}$$

6 $${v}_{sq}={R}_{s}{i}_{sq}+{\omega }_{s}{\lambda }_{sd}+\frac{d{\lambda }_{sq}}{dt}$$

7 $${v}_{rd}={R}_{r}{i}_{rd}-{(\omega }_{s}{-\omega }_{r}){\lambda }_{rq}+\frac{d{\lambda }_{rd}}{dt}$$

8 $${v}_{rq}={R}_{r}{i}_{rq}+{(\omega }_{s}{-\omega }_{r}){\lambda }_{rd}+\frac{d{\lambda }_{rq}}{dt}$$

### PV system model

A PV array comprises of 5 PV sections associated in a series to shape a PV string. 66 PV strings are associated in a shunt to obtain the optimal power of 100 kW. A Sun Power (SPR-305E-WHT-D) panel is implemented in this system. The single diode model is the most utilized. PV circuit output current can be illustrated (9–10)^53–55^:

9 $${I}_{PV}={I}_{Ph}-{I}_{D}-{I}_{P}$$

10 $${I}_{PV}= {I}_{Ph}- {I}_{s}\cdot \left[exp \left(\frac{q\left({V}_{PV}+{R}_{s}{I}_{PV}\right)}{{A}_{c}{K}_{B}T}\right)-1\right] -\left(\frac{{V}_{PV}+{R}_{s}{I}_{PV}}{{R}_{P}}\right)$$

### DC/AC inverter

**Figure 3:**
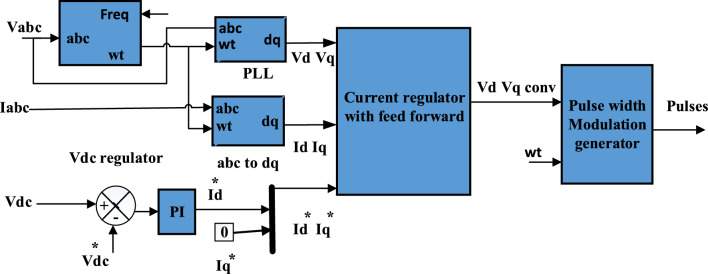
Control system of DC/AC converter.

As shown in Fig. 3, the fundamental components of the inverter are phase locked loop (PLL), DC-link voltage, and current regulator controllers, all of which are associated together to keep DC voltage at its reference value and manage reactive power. A PLL produces the egrid voltage angle ($${\theta }_{Pll}$$), which changes the (abc) value to the axis (d–q). The DC voltage controller keeps DC voltage at its rated value of 500 V by comparing the actual DC voltage $${(v}_{dc})$$ with the reference value ($${v}_{dc}^{*})$$ and the difference is applied to the PI-controller to determine rated current in d-axis ($${I}_{d}^{*}$$). Current regulator based feed forward get ($${V}_{d conv ,}{V}_{q conv}$$) to pulse width generator to get pulses for inverter. The feed forward method is simple and readily realized in the traditional control structure. It can eliminate the negative damping of inverter impedance in a wide frequency range. It contributes in eliminating the disturbance in the current control loop and improve the system stability under various working conditions^56^. Voltage equations in the d–q reference are as follows^37^:

11 $${V}_{d\_conv}={v}_{d}+{R}_{f}{I}_{d}-{L}_{f}{I}_{q}+{l}_{f}\frac{d{l}_{d}}{dt}$$

12 $${V}_{q\_conv}={v}_{q}+{R}_{f}{I}_{q}+{L}_{f}{I}_{d}+{l}_{f}\frac{d{l}_{q}}{dt}$$

### DC/DC converter

**Figure 4:**
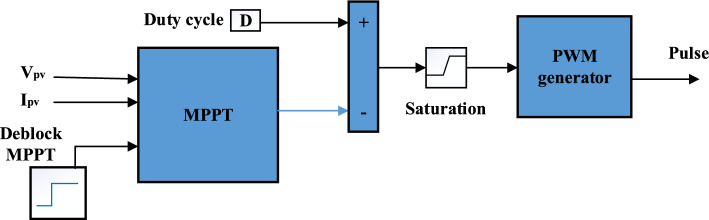
Control system of DC/DC converter.

A boost converter plays a significant role in transferring the solar power to the DC/AC inverter by regulating MP through an incremental conductance MPPT controller^57,58^. It regulates PV voltage through an IGBT switch, who’s on-to-off time with a constant frequency is known as the duty cycle as shown in Fig. 4. The duty cycle is calculated by the following equations:

13 $${V}_{dc}=\frac{{V}_{PV}}{1-D}$$

14 $$\frac{d{I}_{L}}{dt}=\frac{1}{l}\left({V}_{PV}-R{I}_{L}-(1-D){V}_{dc}\right)$$

15 $$\frac{d{V}_{PV}}{dt}=\frac{1}{{C}_{PV}}\left({I}_{PV}-\frac{{I}_{dc}}{(1-D)}\right)$$

16 $${I}_{dc}={I}_{L}(1-D)$$

### ***Model of ***bidirectional*** DC/DC converter***

**Figure 5:**
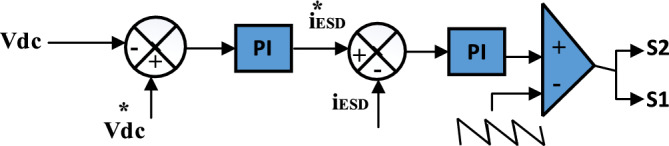
Control system of bidirectional DC/DC converter.

Figure 1c demonstrates the bidirectional DC/DC converter that is utilized to add a battery or the SC into the DC-link to obtain the DC-bus voltage at a regulated value. Controlling a DC/DC converter can be achieved by two loops. A PI-controller is utilized in the outer control loop to generate the reference current. As indicated in Fig. 5, the inner control loop is utilized to generate the pulse signal to switch on ${S}_{2}$_._ ${S}_{1}$ is turned off (boost mode) to discharge electrical energy to the DC-link to power the ESD. During a fault condition, ESD is performed regardless of which switch (${S}_{1}$) is on or (${S}_{2}$) is off (buck mode) to store excess power in the DC-link to keep the DC-link balanced and return it when needed^59^. The bidirectional DC/DC converter's dynamic average model's mathematical formulae are as follows:

17 $${i}_{ESD}^{*}=\left(kp+\frac{KI}{s}\right){(V}_{dc}^{*}-{V}_{dc})$$

18 $${d}_{b}=\left(kp+\frac{KI}{S}\right)({i}_{b}^{*}-{i}_{b})$$

### Crowbar scheme

CB is a vital protection scheme utilized to safeguard DFIG during a fault. It is composed of a resistor connected to a full-bridge rectifier, which is connected directly to the rotor circuit. By dampening excess power, CB prevents damage to the DFIG during a fault. When CB is online, DFIG is similar to a squirrel cage induction generator during the CB circuit. With a low resistance value, the electromagnetic torque increases, while a higher value increases the ripple voltage in the DC-link. The optimal value of CB resistance is calculated by (19)^60–62^.

19 $${R}_{cr}=\frac{\surd 2({v}_{max}{\omega }_{s}{L}_{S})}{\surd \left(3.2{v}_{s}^{2}-2{v}_{max}^{2}\right)}$$

### DC crowbar scheme for PV system and its modelling

This scheme is proposed to protect the PV inverter from increasing voltage and maintain DC voltage at its rated value by absorbing the overvoltage to achieve the LVFRT requirement of the egrid code during a disturbance. The DC CB is associated to the DC-link in parallel and managed by an IGBT switch. The estimation of the CB resistance must be calculated carefully since the PV inverter performance is sensitive to the fault. Dc voltage should be checked between the specified high grid voltage “${v}_{g}$ = 1.1 pu” and the low grid voltage ${v}_{g}$ of 0.85 pu^63^. The ideal estimation of CB resistance is determined by the following equations:

20 $$\Delta {v}_{dc}= {v}_{dc}^{max}-{v}_{dc}$$

21 $${R}_{CB}=\frac{\Delta {v}_{dc}}{\Delta {I}_{in}}$$

22 $$IV- {I}_{inv}{v}_{inv}=\Delta {p}_{dc}$$

23 $$\Delta {p}_{dc}={C}_{dc}{v}_{dc}\frac{d{v}_{dc}}{dt}$$

### Battery modeling

The battery is the most widely used ESD due to its ability to charge and discharge electrical power through a bidirectional DC/DC converter to increase the reliability of the system as PV and wind systems are dependent on the weather^49^. The high efficiency of a battery can be kept by controlling its state of charge (SoC). Various types of rechargeable batteries are used, but lithium-ion is chosen based on decision matrix (DM) criteria in^64,65^. Its size can be calculated by following (24–26), which describes the discharge and charge statutes of batteries. Battery power is described in (27)^39^.

24 $$Capacity\,\left(Ah\right)=\frac{Total\, load \,power\,\left(W\right)\times \,Time\,(h)}{\,DOD\times voltage \,(v)}$$

where DOD is the depth of discharge

25 $${V}_{b}={V}_{g}-{R}_{b}{i}_{b}-K\frac{q}{q-{i}_{t}}{(i}_{t}+{i}_{b}^{*})+A{e}^{-B{i}_{t}}$$

26 $${V}_{b}={V}_{g}-{R}_{b}{i}_{b}-p\frac{q}{{i}_{t}-0.1q}{i}_{b}^{*}-p\frac{q}{q-{i}_{t}}{i}_{t}+A{e}^{-B{i}_{t}}$$

27 $${p}_{BS}={V}_{b}{i}_{b}-{R}_{b}{i}_{b}^{2}$$

### Supercapacitor modelling

To solve issues of LVFRT capability, SC is associated with the DC-link of the renewable system through a bidirectional DC/DC converter. It has a high-power density, a long lifetime, a wide temperature range, and a high response time to charge and discharge energy, but only stores energy for a short time^66^. Equations (28) and (29) are used to define the proper size of SC in steady-state operation^5^. On the other hand, Eq. (30) provides a SoC expression that can be used to save the capacitor lifetime.

28 $$\Delta E=0.2{p}_{nominal}\Delta t$$

29 $$\Delta E=\frac{1}{2}C({V}_{max}^{2}-{V}_{min}^{2})$$

30 $$\frac{d}{dt}SoC=\frac{1}{{C}_{SC }{V}_{maxsc}}isc$$

## Results and discussions

### PV system associated to egrid

**Figure 6:**
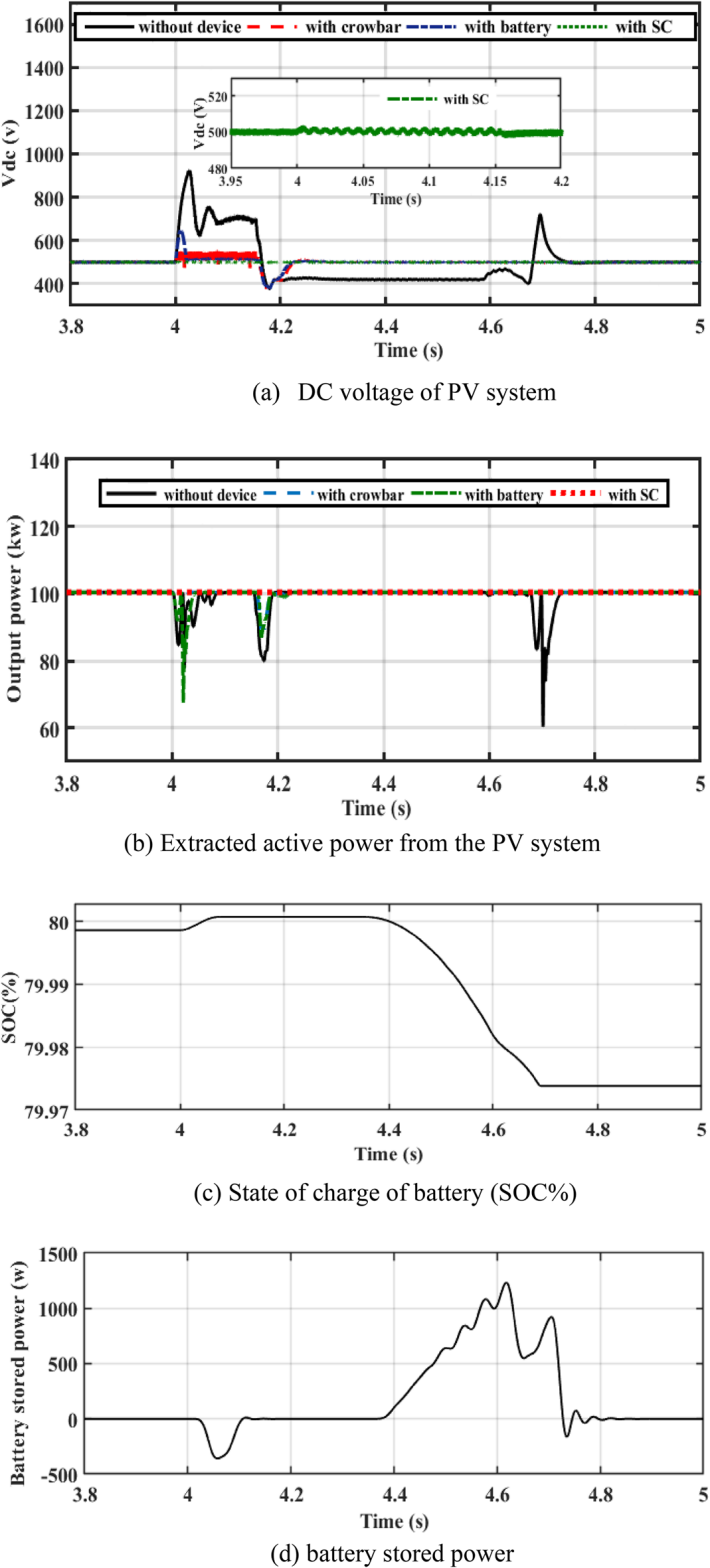
Performance of the PV system.

As shown in Fig. 6, PV associated to the egrid with CB, battery, and SC is studied. The performance of a PV system with the three devices is studied under symmetrical fault (3ph fault) and asymmetrical fault (2LG fault) for 150 ms.

### Performance of PV system under symmetrical fault

The PV system associated to the egrid during a 3 ph fault is analyzed. Figure 6a depicts the DC bus voltage without any devices, which fluctuates between 910 and 390 V. After adding the CB circuit, it fluctuated between 380 and 550 V. After adding the CB circuit, it fluctuated between 380 and 550 V. After the battery is added, the DC bus voltage oscillates between 600 and 390 V, then recovers to its reference voltage at the specified level of 500 V. A closer look at Fig. 6a reveals that SC keeps the DC voltage at the specified level of 500 V and contributes to quickly recovering the DC bus voltage to the specified level.

By adding CB, the DC bus voltage is higher than when the battery is added to the DC-link voltage. As revealed in Fig. 6b, active power extracted from the PV system oscillates, then the fault reduces the power. When the CB is added, power fluctuation decreases to 89 kW. When a battery is added, power oscillation is decreased to 69 kW. By adding SC, power oscillation is reduced to 98 kW. The oscillations are stabilized at 89 kW after the fault is resolved, and the active power does not exceed the reference value, as cleared in Fig. 6b. It may establish that the SC successfully reduces the oscillations of active power and maintains it at its predefined value. The battery charges during the fault duration as shown by Fig. 6c,d to support the DC-link voltage.

**Figure 7:**
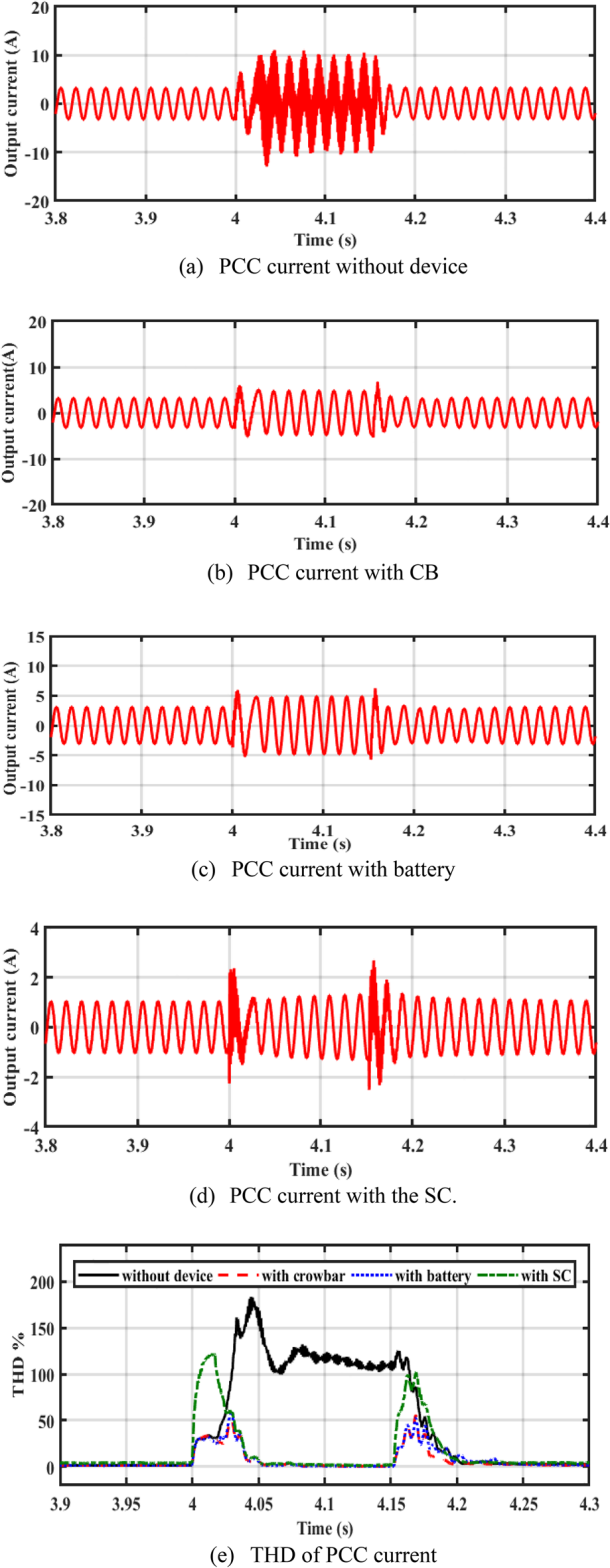
Performance of the system at PCC.

In Fig. 7a–d, it is observed that the current in the cases of CB, battery, and SC is lower than the rotor current during a fault without device (reduces the current to 3 A). Figure 7e shows the total harmonic distortion (THD) of PCC current without device, with CB, battery, and SC. The THD is about 2.6% before and after the fault. THD during fault is more enhanced with adding of energy storge devices (ESDs).

#### Performance of PV system under unsymmetrical fault

**Figure 8:**
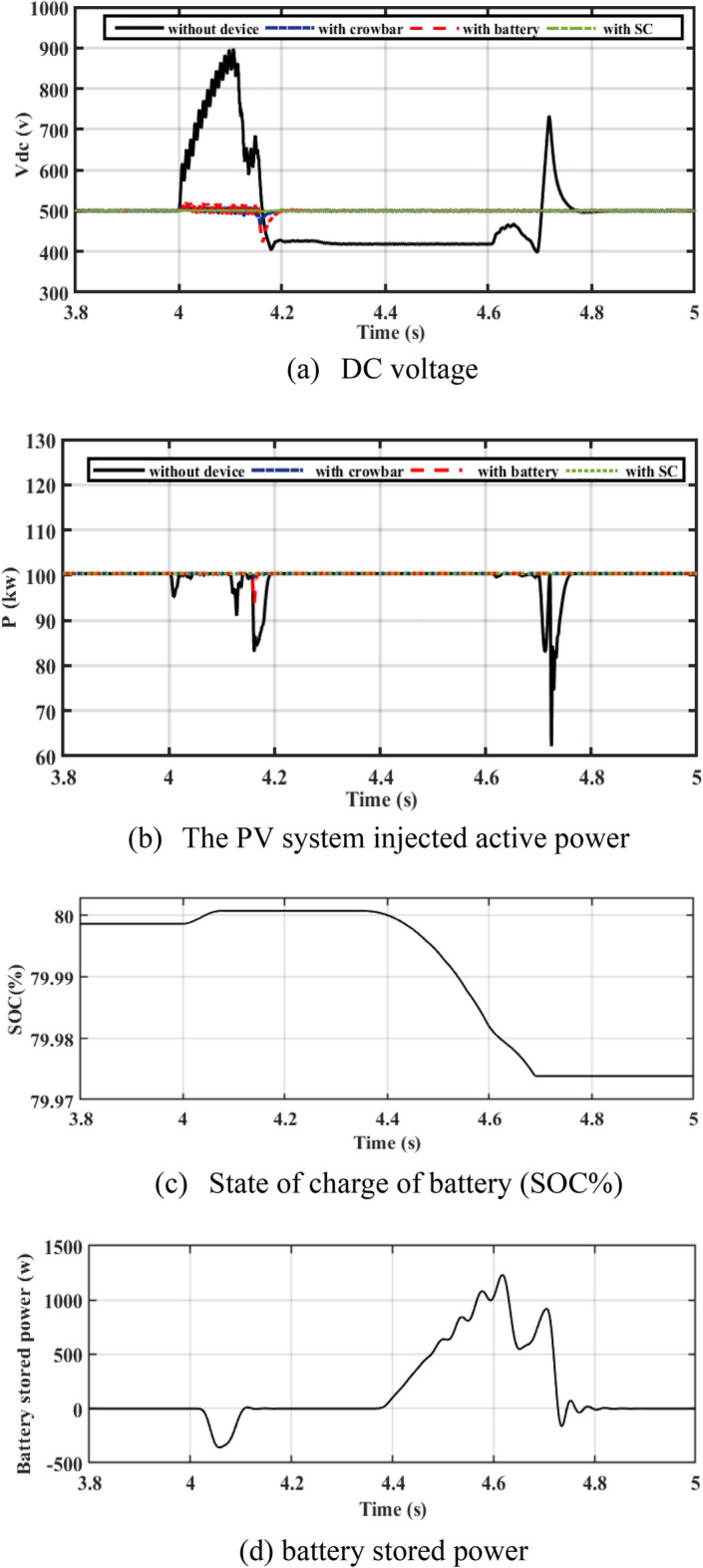
Performance of the PV system during unsymmetrical fault (2LG).

A PV system on egrid mode is tested when a severe 2LG fault is applied. In Fig. 8a, the DC voltage changes between 900 and 400 V and it increases rapidly during fault without control device. As a result of adding the CB circuit, it fluctuated between 430 and 530 V and then returned to the rated value of 500 V again after clearing the fault for 0.2 s. In the case of battery connection, the DC voltage varies between 570 and 480 V. The SC keeps DC voltage at its rated level.

Figure 8b shows active power where the power drops to 62 kW. After adding CB, the power oscillation is reduced to 95 kW. The power is reduced to 98 kW with the battery, while the SC stabilizes the fluctuations of power at a rated value. The battery charges during the fault duration as shown by Fig. 8c,d to support the DC-link voltage.

**Figure 9:**
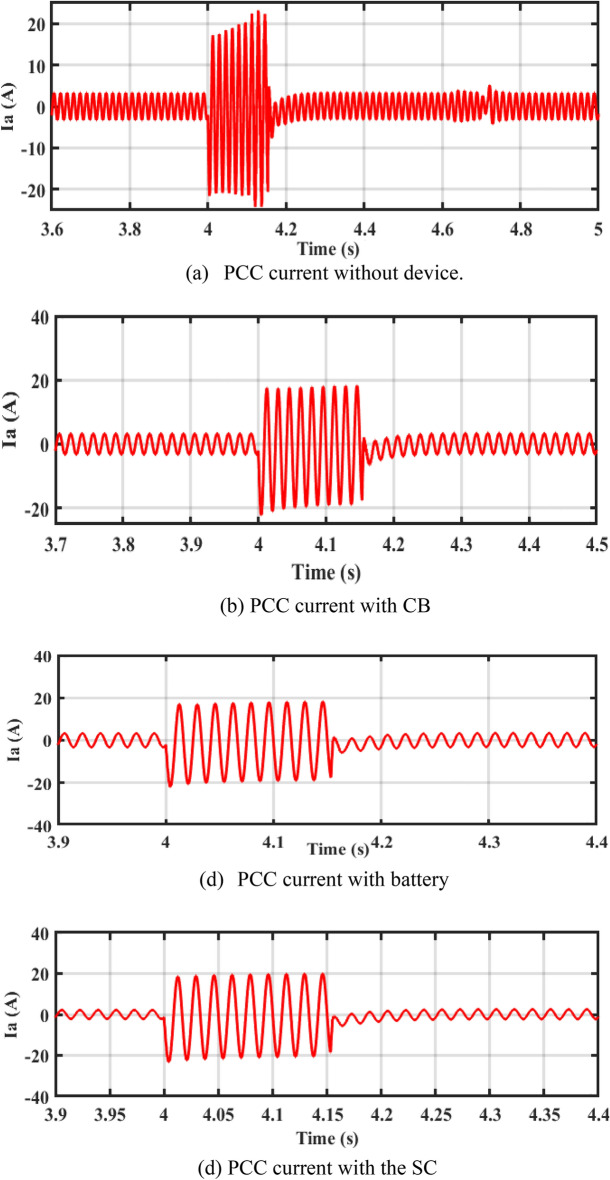
Performance of the PV system during unsymmetrical fault (2LG).

The output current of the CB circuit is less than the current of the battery and SC in Fig. 9a–d.

### eGrid-connected DFIG based wind turbine

The performance characteristics of a 6 MW DFIG-WT farm are studied in a MATLAB/Simulink environment with different LVFRT schemes (CB, battery, and SC). The wind farm operates at an 11 m/s fixed wind speed.

#### Performance WT system under symmetrical fault

**Figure 10:**
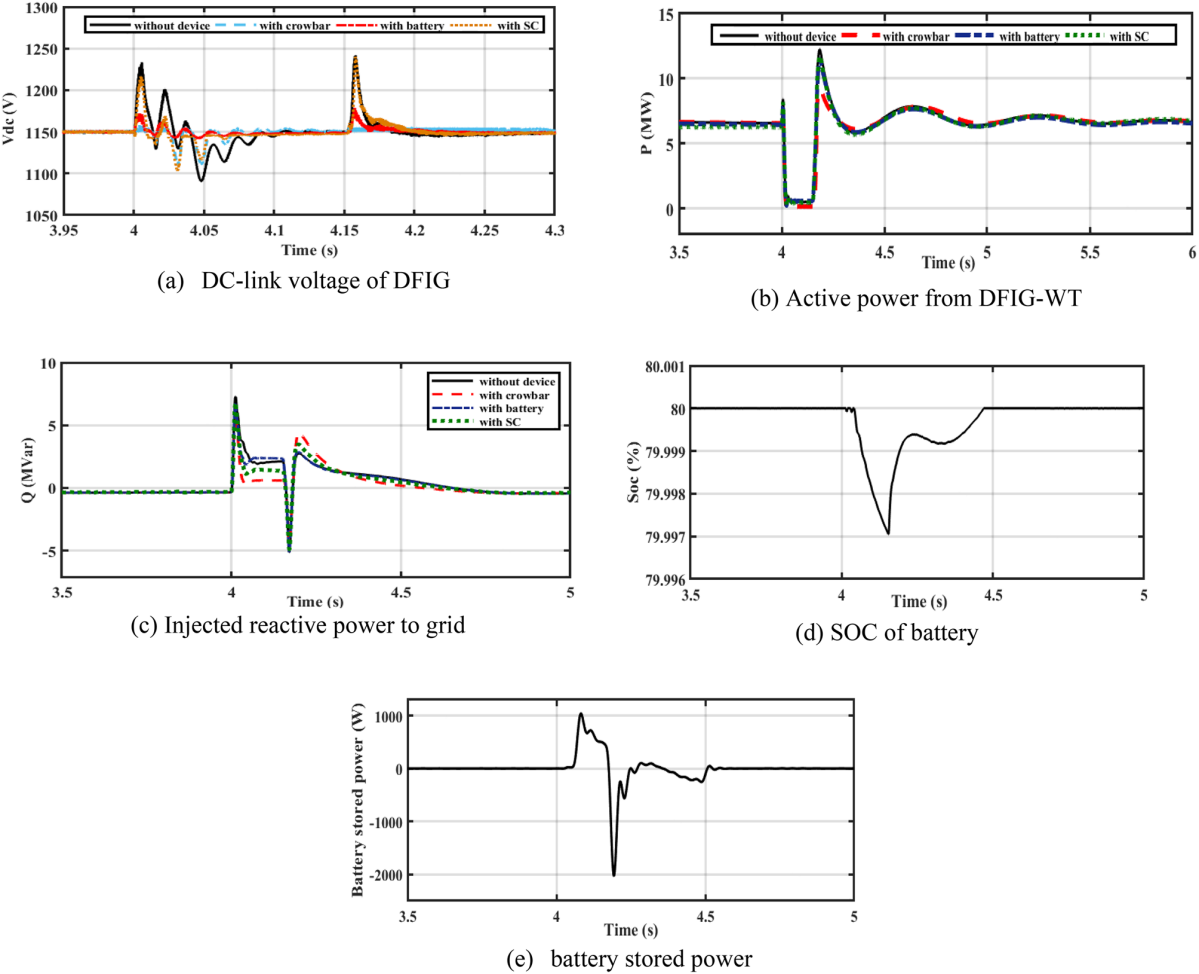
Performance of DFIG-WT on-grid mode during the symmetrical fault.

The performance of the DFIG-WT on-egrid system with CB, batteries, and SC is depicted in Fig. 10. The 3ph fault is implemented in PCC at 0.4 s and lasts 150 ms. The SOC of the battery is considered below 80%. Figure 10a demonstrates that the battery maintains the DC bus voltage better than SC. When the SC is used, oscillations in active power decrease (see Fig. 10b). Figure 10c shows that a CB extracts more reactive power than the battery case. The battery charges during the fault duration as shown by Fig. 10c,d to support the DC-link voltage.

**Figure 11:**
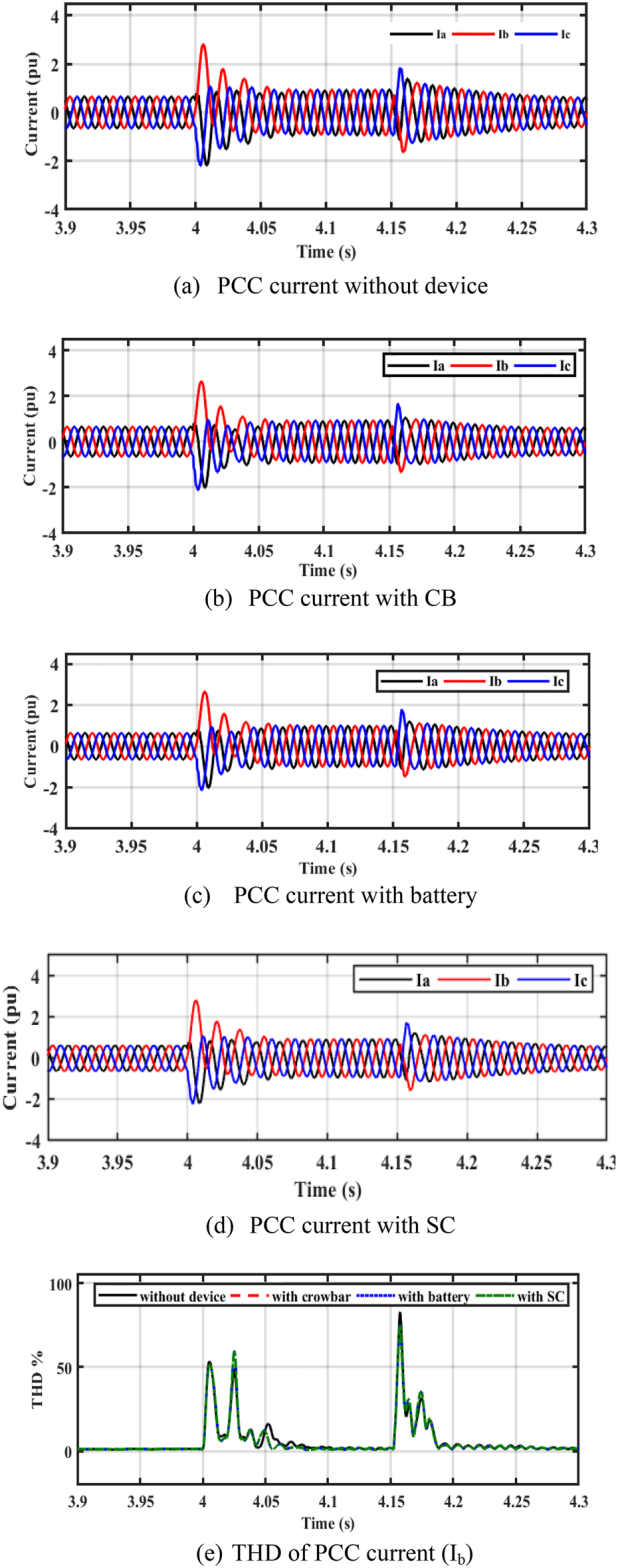
Performance of DFIG-WT on-grid mode during symmetrical fault.

In Fig. 11a–d, it is observed that the rotor currents in the cases of CB, battery, and SC is lower than the currents during a fault without device. Figure 11e shows the THD of PCC current without device, with CB, with battery, and SC. The THD is about 2.6% before and after the fault. THD during fault is more enhanced with adding of ESDs.

#### Performance of WT system under asymmetrical faults

**Figure 12:**
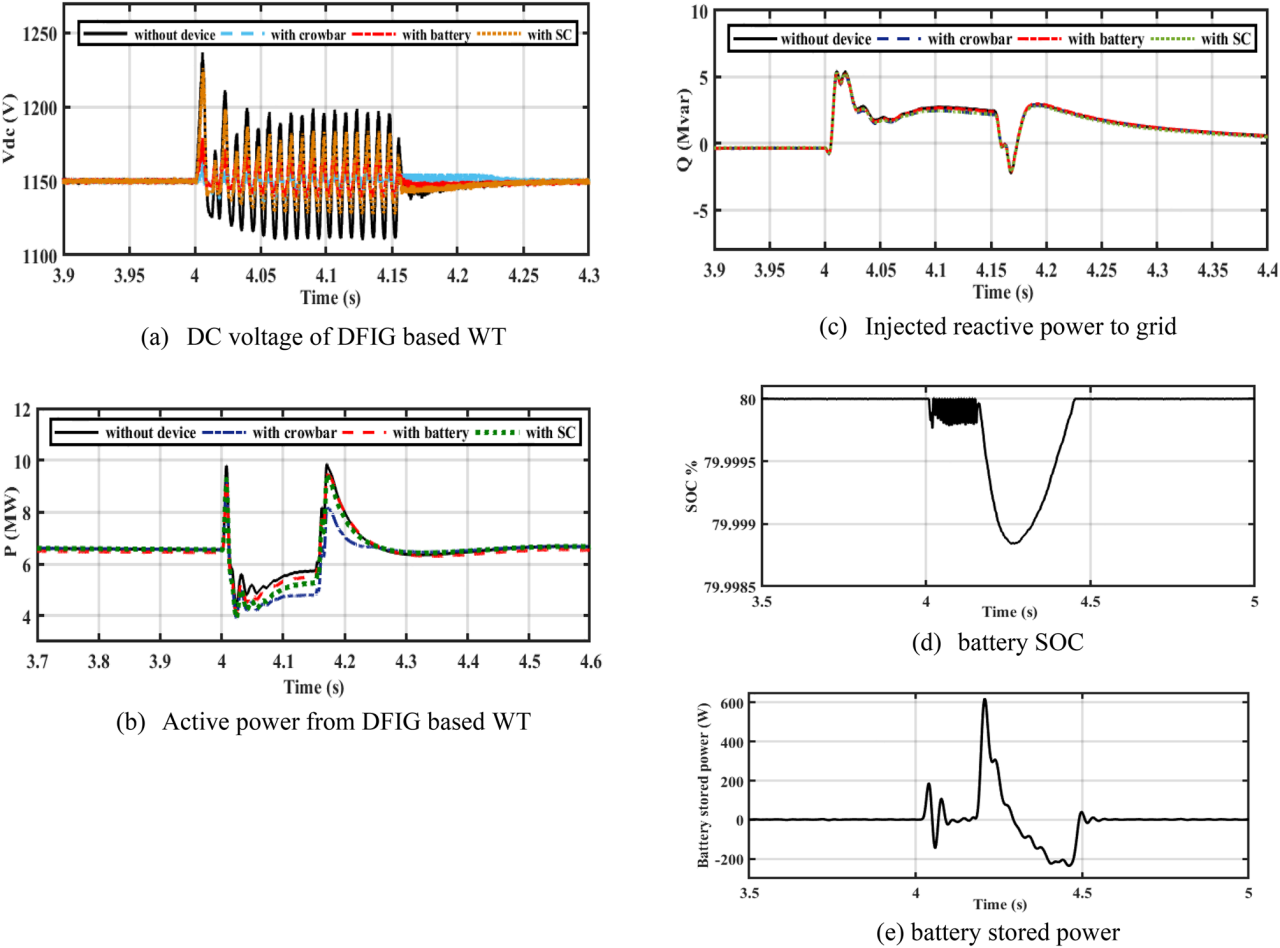
Performance of DFIG-WT on-grid mode during unsymmetrical fault.

The performance of the DFIG-WT on-egrid system is analyzed with CB, battery, and SC. 2LG is applied at PCC at 0.4 s and continues for 150 ms. The SOC of a battery is considered below 80% which is shown in Fig. 12d. As revealed in Fig. 12a, CB reduces dc voltage ripples to 1155 V during the fault duration. During the fault duration, the battery reduces dc voltage to 1157 V, as shown in Fig. 12a. When compared to other schemes, Fig. 12a demonstrates that the battery improves the ripples in the DC bus voltage during an issue duration. The battery charges during the fault duration as shown by Fig. 12d,e to support the DC-link voltage. As seen in Fig. 12b, oscillations of active power are decreased because of adding SC. In Fig. 12c, CB extracts more reactive power than the battery.

**Figure 13:**
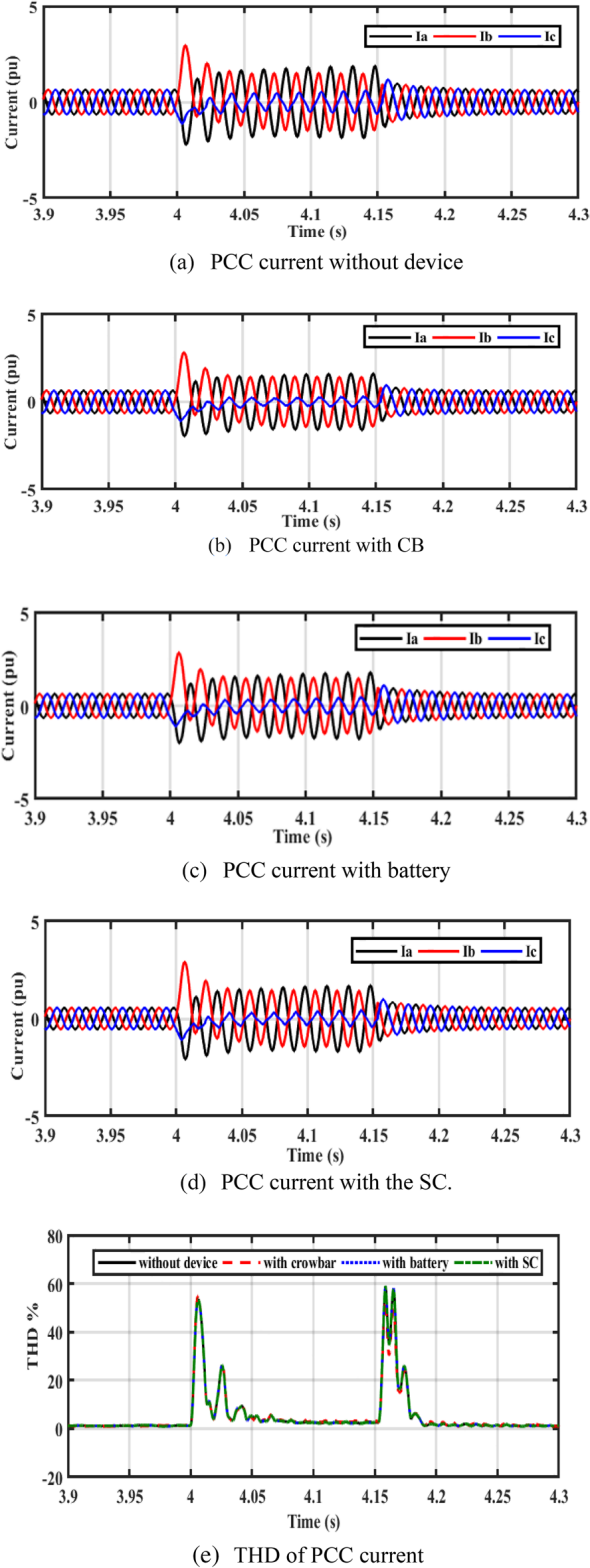
Performance of DFIG-WT on-grid mode during the asymmetrical fault.

By closer look to Fig. 13a–d, the performances of DFIG-WT on-grid mode during the asymmetrical fault with and without adding any storage as illustrated. Figure 13e shows the THD of PCC current without device, with CB, with battery, and SC. The THD is about 2.6% before and after the fault, the THD during fault is more enhanced with adding of ESDs.

### Grid-connected hybrid power system PV/WT

The PV/WT system on-grid mode is shown in Fig. 1c. In steady state, the PV system produces 400 kW while the wind turbine produces 1 MW. During a 3-ph fault, the DC-link voltage is dropped, and the grid power is decreased. Hybrid power systems' performance is analyzed and compared with different LVFRT schemes. LVFRT capability schemes protect the rotor circuits and the dc-bus voltage from overvoltage. An investigation of the effectiveness of various LVFRT schemes on the PV/WT hybrid power system is discussed during a 3-ph fault and a 2L to G fault lasting for 150 ms at t = 4 s.

#### Performance of hybrid PV/WT system under balanced fault.

**Figure 14:**
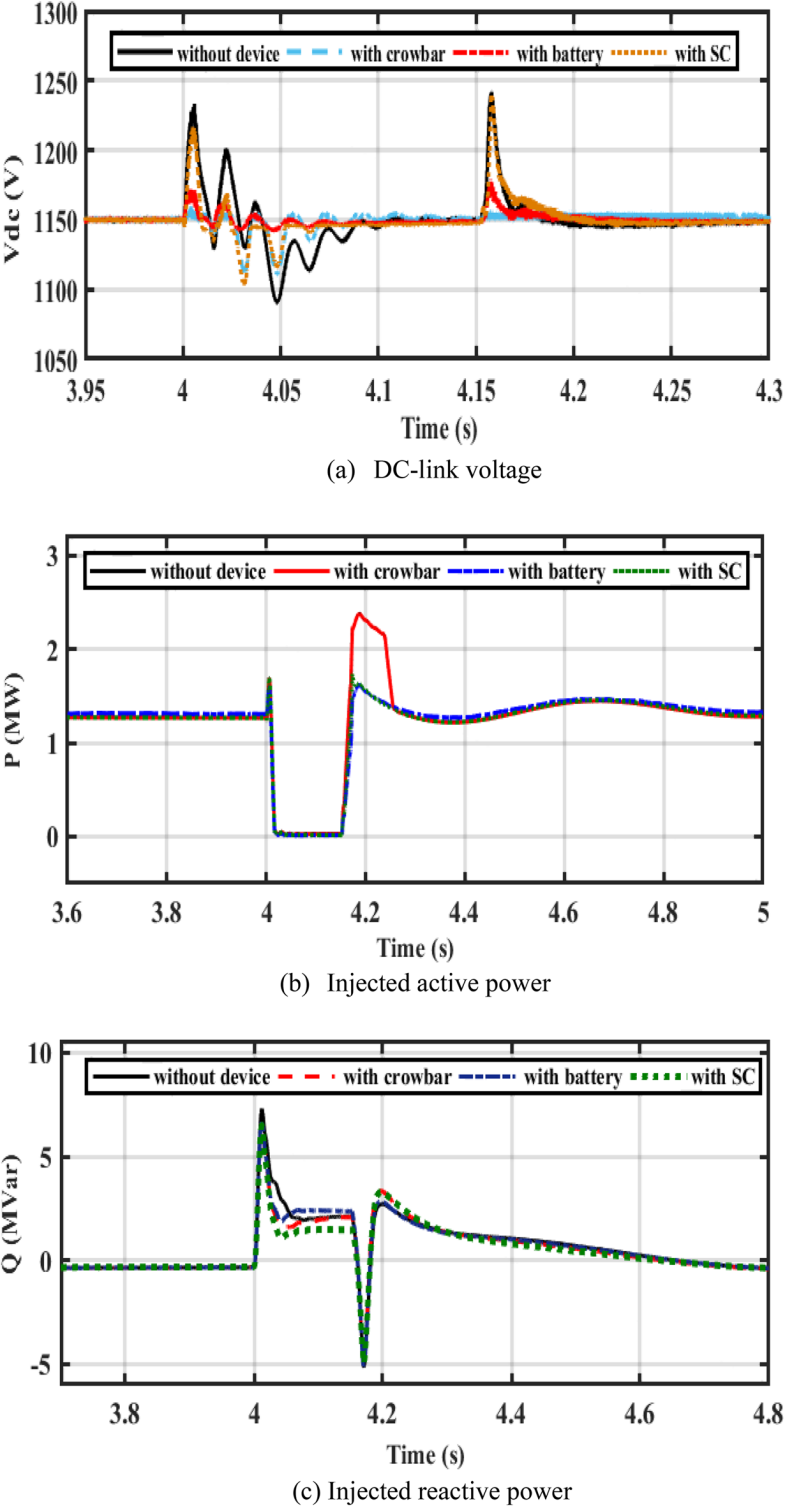
Performance of the PV/WT on-grid mode.

Figure 14 illustrates the performance of a hybrid PV/WT system with LVFRT devices (CB, battery, and SC). Figure 14a demonstrates the DC bus voltage during a three-phase fault. If there is no device, DC voltage increases dramatically to 1240 V and there is a significant overshoot. If a CB circuit is added, the DC voltage decreases from 1240 to 1170 V. After adding a battery, DC voltage decreases and regains to its reference level with low oscillations during the fault period. The figure also shows that SC preserves the DC bus voltage constant at its rated value of 1150 V during an issue. SC keeps DC voltage at its rated level, decreases oscillations of active power, and the system reaches stability faster. Figure 14b resolves active power delivered to the egrid.

**Figure 15:**
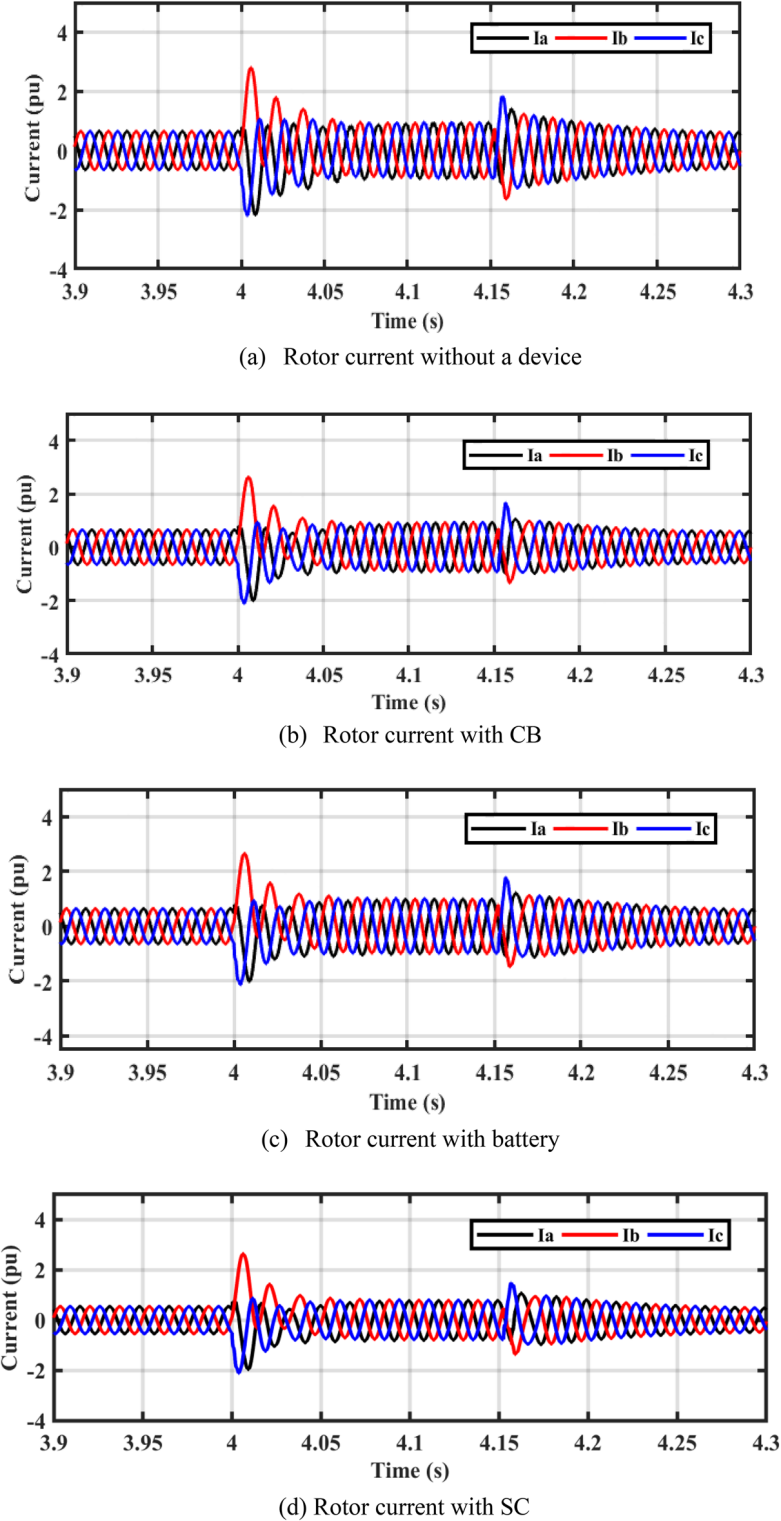
Performance of PV/WT on-grid mode.

In steady-state, the PV unit injects 400 kW of power into DC bus voltage using the incremental conductance MPPT technique, and the DFIG-WT injects 1 MW into the grid. A three-phase fault decreases active power, which increases the DC voltage. When LVFRT schemes are used, active power oscillations are decreased. The battery stores power, but the power is dissipated in CB resistance. According to Fig. 14b, after a fault has been cleared, the osculation of active power decreases with the LVFRT schemes. As demonstrated in Fig. 14c, SC absorbs less reactive power. As shown in Fig. 15, the CB, battery, and SC contribute to reducing the rotor current during a fault.

#### Performance of hybrid PV/WT system under asymmetrical fault

**Figure 16:**
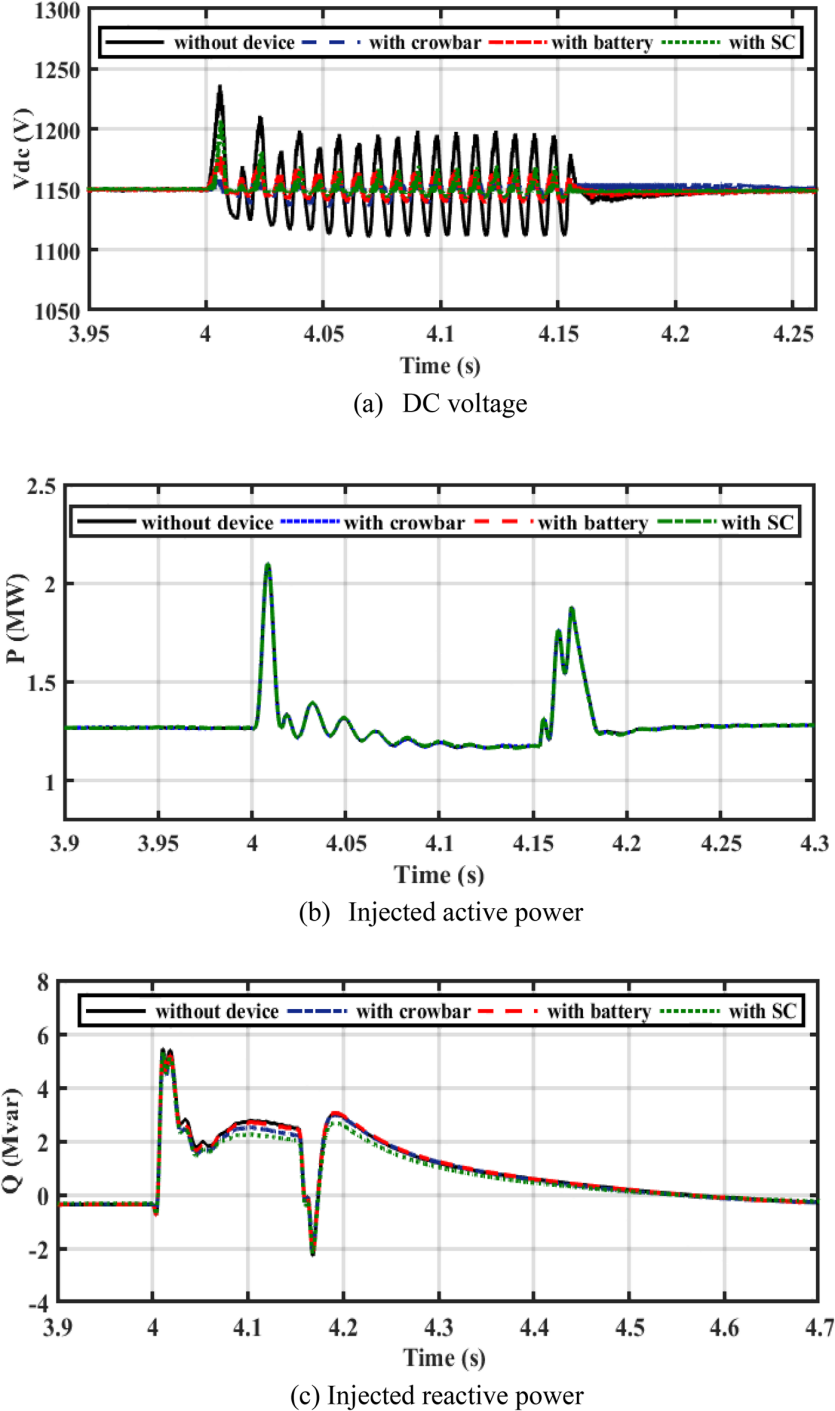
Performance of the hybrid PV/WT during 2LG fault.

Figure 16 shows the performance of PV/WT on-grid mode with three different LVFRT strategies (CB, batteries, and SC). Figure 16a illustrates the DC bus voltage during a 2L to ground fault. As a result of a 2L to ground fault, DC voltage increases drastically, and a large overshoot occurs. By adding CB, the DC voltage decreases to 1160 V. As a result of adding the battery, the DC-link voltage decreases to 1157 V. SC decreases the DC voltage to 1157 V. The SC and the battery, as shown in Fig. 16a, both contribute to lower DC voltage overshoot.

**Figure 17:**
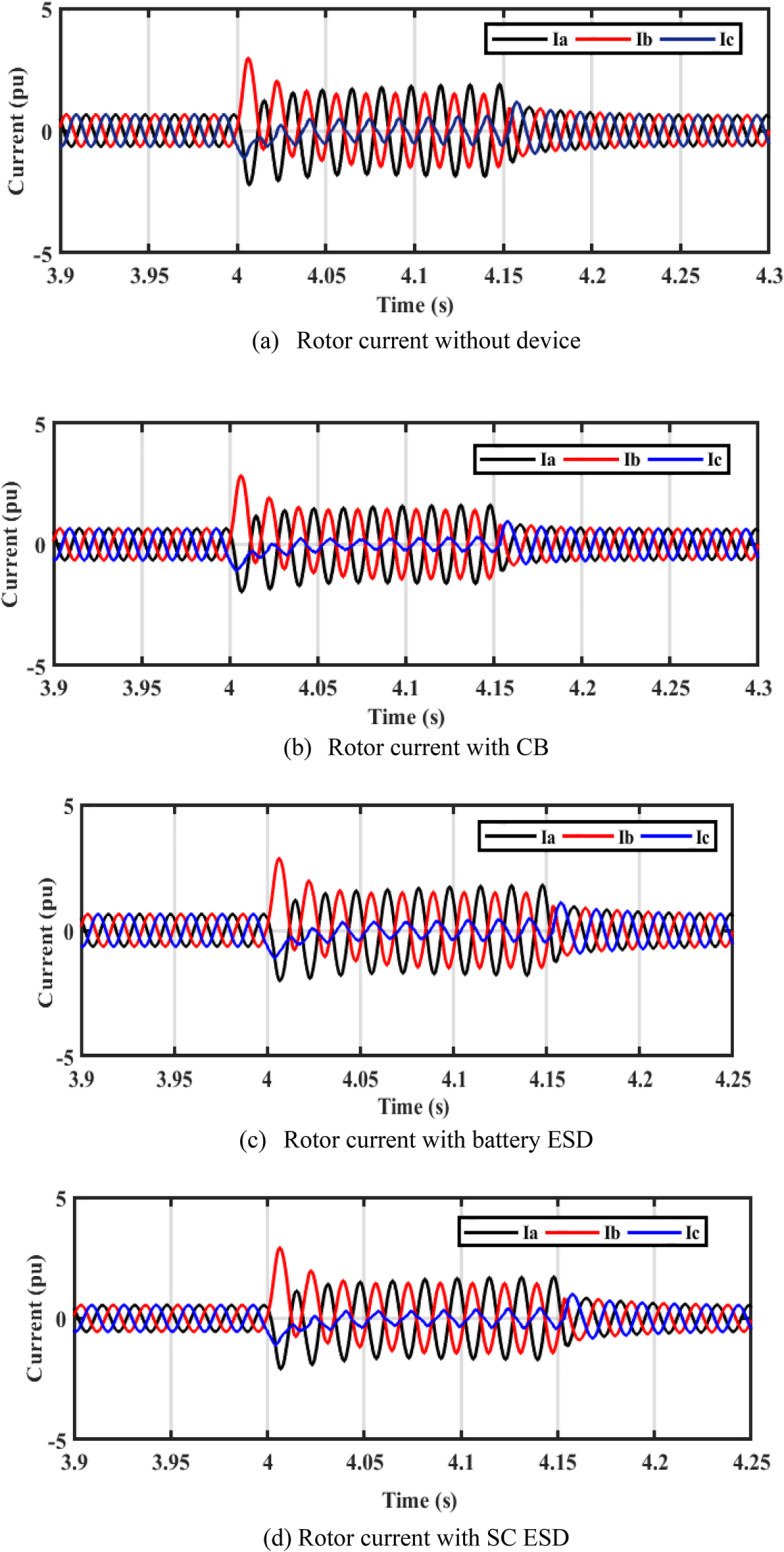
Hybrid PV/WT Performance during 2L to ground.

In a steady-state operation, the PV unit injects the MP of 400 kW into the DC bus voltage by using the incremental conductance MPPT technique, and the DFIG-based WT injects 1 MW into the grid. During a 2LG fault, the power decreases and the DC-bus voltage is increased. When LVFRT schemes are added to the DC-link of the power system, oscillations in active power are decreased. By adding CB, active power increases slightly. When the battery is implemented, active power will increase slightly as compared to the CB circuit. Power is stored in batteries but is dissipated in CB resistance. Based on Fig. 16b, CB reduces the overshoot of active power during and after the fault has been fixed. The absorbed reactive power, as with SC, is lower than with other LVFRT methods, as shown in Fig. 16c. As demonstrated in Fig. 17, CB, battery, and SC contribute to reducing the rotor current during faults.

## Procedures of the CBO with problem formulation

The intelligent optimization algorithms may be used to improve the LVFRT capability of the studied systems. The ratings of both the battery and the super capacitor have a great effect on the DC bus voltage. In our research, the CBO is used to determine the suitable ratings of both the battery and the super capacitor according to the minimum ripples in the DC-link voltage.

Like other intelligent optimization methods, CBO follows the behavior of a swarm of birds in the sea or lake^67,68^. The advantages of CBO are that it searches volatile and multi-dimensional solution spaces and finds the optimal answers quickly. It emulates two different actions of birds on the water surface; the first is infrequent and the second is frequent. To find food, swarms move towards a group of leaders. However, the end of the swarm forms a chain of coots, each of which passes behind its predecessors. Next, the algorithm is applied to several test functions. A CBO is assessed based on 13 criterion functions (with 30, 100, and 500 dimensions). Where Dim acts the function dimensions, range is the boundary of the search space of the function and is the optimal value. The coot’s leaders are viewed as a percentage of the total assumed coot population, ${N}_{coots}$, and the rest are the coot’s followers. The positions of followers (${pos}_{coots}$) and leaders (${Fit}_{coots}$) are randomly selected as expressed in (31) and (32), respectively.

31 $${pos}_{coots}={Rand}_{coot}.\left({U}_{b}-{L}_{b}\right)+{L}_{b}$$

where ${U}_{b}$ is the major limit, and ${L}_{b}$ is the minor limit. The fitness of all Coot’s followers ${Fit}_{coots}$ can be calculated by means of the objective function (${F}_{obj}$) as per (32). The best global coots score ${Gbest}_{score}$ and its position ${Gbest}_{pos}$ are defined by (33).

32 $${Fit}_{coots}\left(1,i\right)={F}_{obj}\left({Pos}_{coot}\left(i\right)\right),i\in {N}_{coots}$$

33 $$\begin{aligned} & {\text{If }}Gbest_{score} > Fit_{coots} \left( {1,i} \right) \\ & Gbest_{score} = Fit_{coots} \left( {1,i} \right) \\ & Gbest_{pos} = Pos_{coot} \left( i \right) \\ \end{aligned}$$

Likewise, the fitness of all Coot’s leaders can be calculated via the ${F}_{obj}$ as formulated in (34). The ${Gbest}_{score}$, and its position ${Gbest}_{pos}$ are defined in (35).

34 $${Fit}_{leaders}\left(1,i\right)={F}_{obj}\left({P}_{leader}\left(i\right)\right),i\in {N}_{leaders}$$

35 $$\begin{aligned} & {\text{If }}Gbest_{score} > Fit_{leaders} \left( {1,i} \right) \\ & Gbest_{score} = Fit_{leaders} \left( {1,i} \right) \\ & Gbest_{pos} = Pos_{leaders} \left( i \right) \\ \end{aligned}$$

where ${N}_{leaders}$ is the number of Coot’s leaders = percentage of ${N}_{pop}$ and ${N}_{coots}$ is the number of a COOT’s followers = ${N}_{pop}$ − ${N}_{leaders}$.

Every one of the coot’s followers is created to one of the coot’s leaders according to haphazard function and updates their positions, accordingly, beginning from iteration 2 to the extreme iterations ($Max\_IT$) as depicted in (36) and (37). The new followers’ positions are limited to be within constraints as in (38).

36 $$R=1+2\cdot {Rand}_{coots}$$

37 $$\begin{aligned} & pos_{coots} \left( i \right) = 2 \cdot Rand_{coots} \cdot cos\left( {2.\pi .R} \right) \\ & [pos_{leaders} \left( k \right) - pos_{coots} \left( i \right)] = 2 \cdot Rand_{coots} \cdot Cos\left( {2 \cdot \pi \cdot R} \right) + pos_{leaders} \left( k \right) \\ & \forall {\text{i}} \in N_{coots} {\text{ and k }} \in N_{leaders} \\ \end{aligned}$$

38 $$\begin{aligned} & {\text{If}} pos_{coots} \left( i \right) > U_{b} , \;{\text{then}}, pos_{coots} \left( i \right) = U_{b,} \\ & {\text{If}} pos_{coots} \left( i \right) > L_{b} ,\; {\text{then}}, pos_{coots} \left( i \right) = L_{b,} \\ \end{aligned}$$

where ${Rand}_{coots}$ is the coot’s follower who haphazardly produced the values and ${Rand}_{leaders}$ is the Coot’s leader randomly produced the values. The updated fitness of all Coot’s followers is calculated and compared with its leader's fitness. If the follower's fitness is better than its corresponding leader, the follower becomes a leader and the leader changes to a follower as in (46).

39 $$\begin{aligned} & {\text{If}} Fit_{coots} \left( {1,i} \right) < Fit_{leaders} \left( {1,k} \right),\; then \\ & Fit_{leaders} \left( {1,k} \right) = Fit_{coots} \left( {1,i} \right) \;and \\ & pos_{leaders} \left( k \right) = pos_{coots} \left( i \right) \\ \end{aligned}$$

The leader’s positions are upgraded according to haphazard functions, as in (25), and (26). The best global score and position are defined in (40).

40 $$\begin{gathered} B = 2 - \left( {IT\left( L \right)/Max\_IT} \right) \hfill \\ R = 1 + 2 \cdot Rand_{leader} \hfill \\ \end{gathered}$$

where $IT(L)$ denotes iteration number L and $Max\_IT$ denotes the number of iterations.

41 $${pos}_{leaders}=B\cdot {Rand}_{leaders}\cdot cos\left(2\cdot \pi \cdot R\right)\cdot [{Gbest}_{pos}-{pos}_{leaders}(i)]+{Gbest}_{pos}]$$

42 $$\begin{aligned} & {\text{If }}Gbest_{score} > Fit_{leaders} \left( {1,i} \right), then \\ & Fit_{leader} \left( {1,k} \right) = Gbest_{score} \\ & pos_{leaders} \left( i \right) = Gbest_{pos} \\ \end{aligned}$$

**Figure 18:**
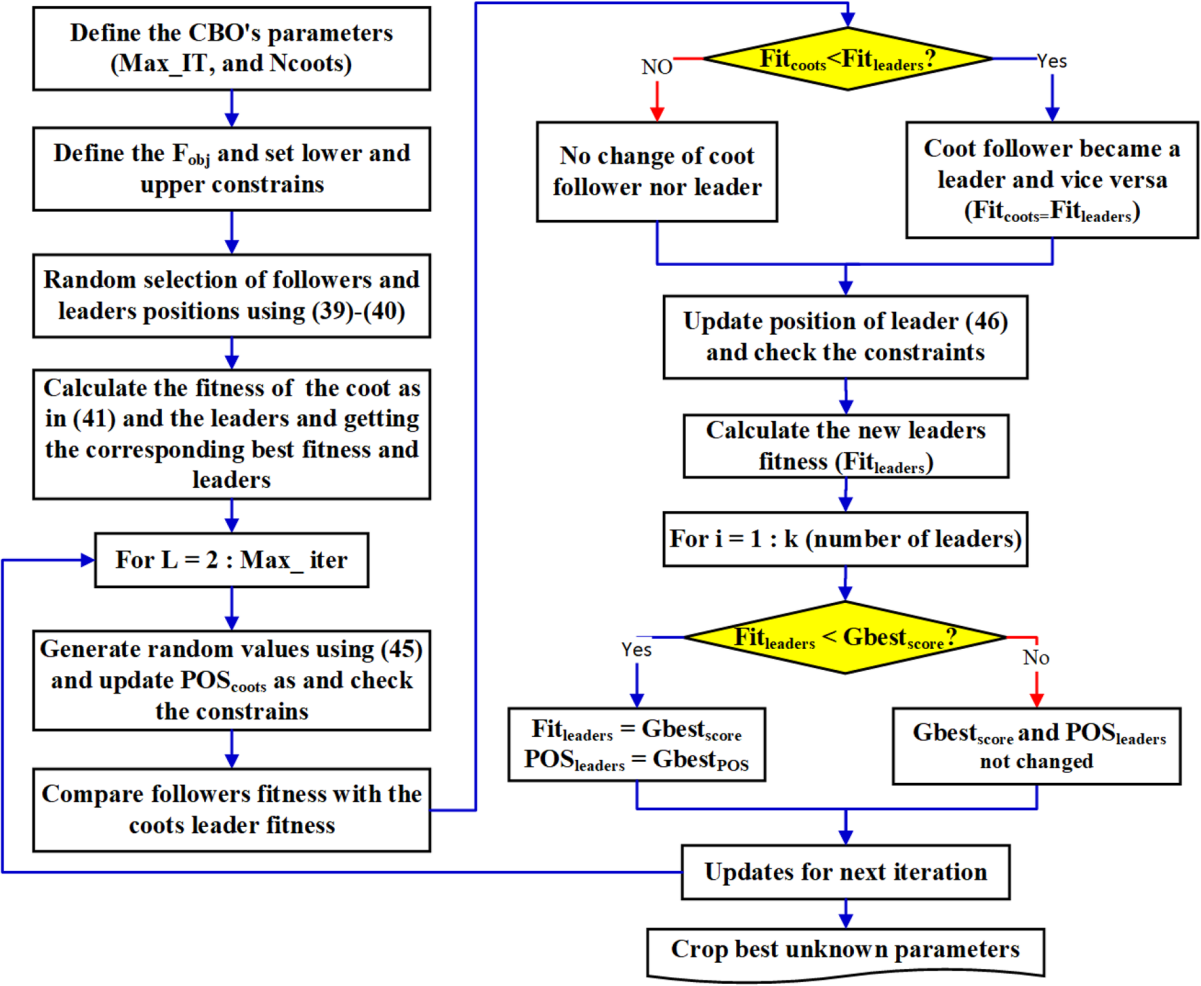
Brief procedures of the CBO.

Further to the above-mentioned, the flowchart shown in Fig. 18 depicts the general procedures of the CBO and detailed instruction^68^.

The objective is to minimize the ripple factor (RF) of the DC-link voltage for three studied systems. The ripples in the DC-link voltage may be calculated from (43).

43 $${F}_{obj}=Min(RF)=\frac{{Vdc}_{max}-{Vdc}_{min}}{{Vdc}_{av}}$$

This objective is subjected to getting the optimal size of battery in Ampere hours and SC (F), the inductor value (H), and tuning the PI-controller of the bidirectional DC/DC converter.

The ${F}_{obj}$ is subjected to the next set of inequality restrictions as pronounced in (44).

44 $$\begin{gathered} 0.1 < C\left( F \right) < 4, 100 < Ah < 1000, 0.00001 < L\left( H \right) < 0.1, \hfill \\ 4 < K_{p} < 45, \;{\text{and }}70 < K_{i} < 400 \hfill \\ \end{gathered}$$

The values of the inductor in (Henry), the battery in (Ah), and the SC in (F) are chosen based on (14), (24) and (29). The min/max limits of the PI-controller are decided by means of numerous trial-and-error procedures.

Demonstrated simulations are executed under the MATLAB/SIMULINK environment and are reinforced by a set of tables and some illustrative figures. The cropped results of the three studied systems using CBO and the calculated ones are compared as depicted in Table 1, cropped overall many independent runs. The calculated ratings of both the battery and the super capacitor are obtained using (24)–(25), and (28)–(29). The values of the parameters of the PI-controller are chosen by the try and error process, while the value of the inductor is calculated based on (14) and (15).

**Table 1:** Comparison between calculated and CBO’s cropped values.

Device	System	Calculated						CBO					
		Ah	C(F)	L(H)	${{\text{k}}}_{{\text{p}}}$	${{\text{k}}}_{{\text{i}}}$	RF	Ah	C(F)	L(H)	${{\text{k}}}_{{\text{p}}}$	${{\text{k}}}_{{\text{i}}}$	RF
Battery (Ah)	PV	500	–	0.03	25	100	0.262	131.1	–	0.00293	19.29	71.77	0.00868
	WT	500	–	1.4	30	200	0.7	250	–	0.0086	15.061	126.117	0.00679
	PV/WT	300	–	1.4	30	200	0.217	172.9	–	0.0485	16.204	135.5	0.00679
Supercapacitor (F)	PV	–	1	0.3	20	150	0.294	–	0.5	0.03782	17.390	75.08195	0.009242
	WT	–	5	1e−3	50	300	0.08	–	3.005	0.00057	31.4	286.0415	0.003432
	PV/WT	–	1	0.01	70	400	0.5	–	0.8587	0.0013	41.3614	372.055	0.11417

**Figure 19:**
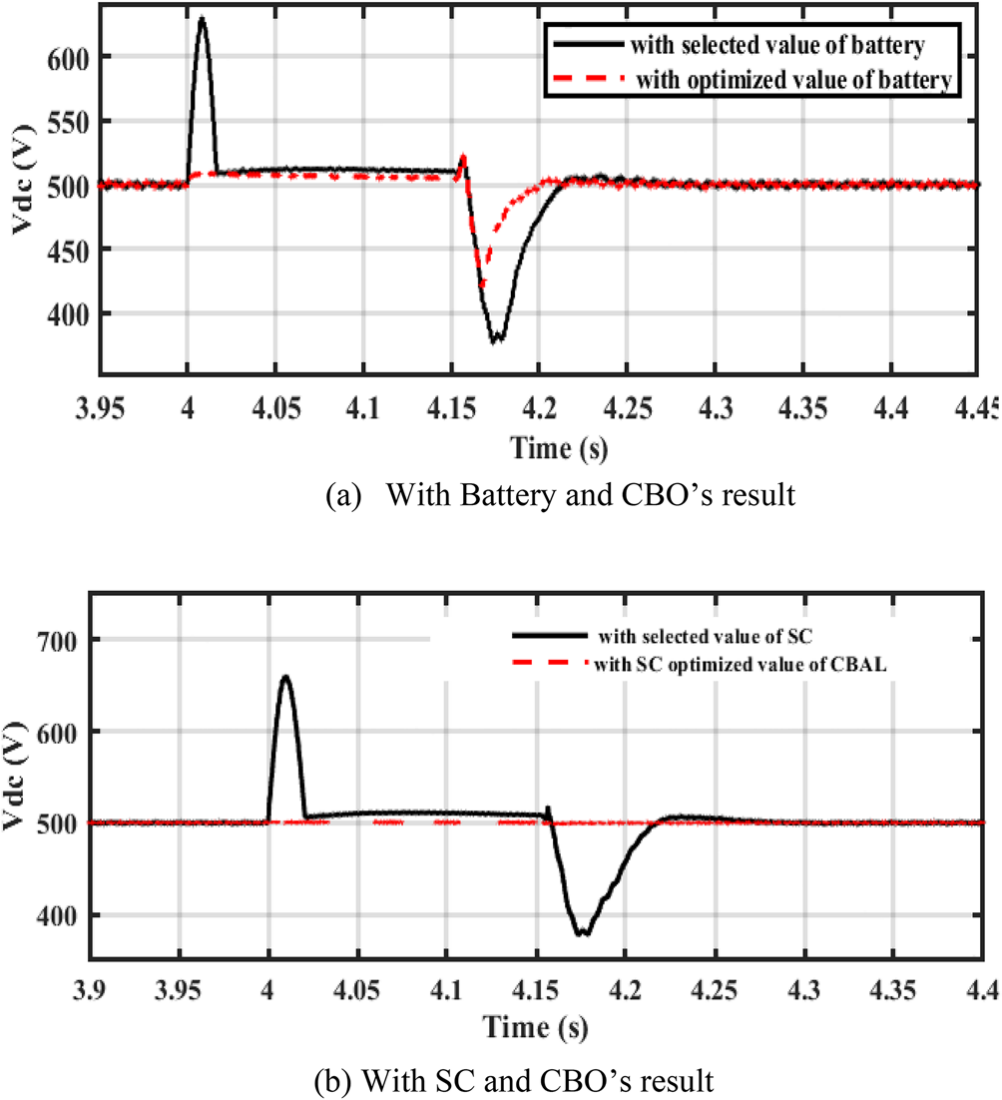
DC-link voltage of PV system associated to the grid.

**Figure 20:**
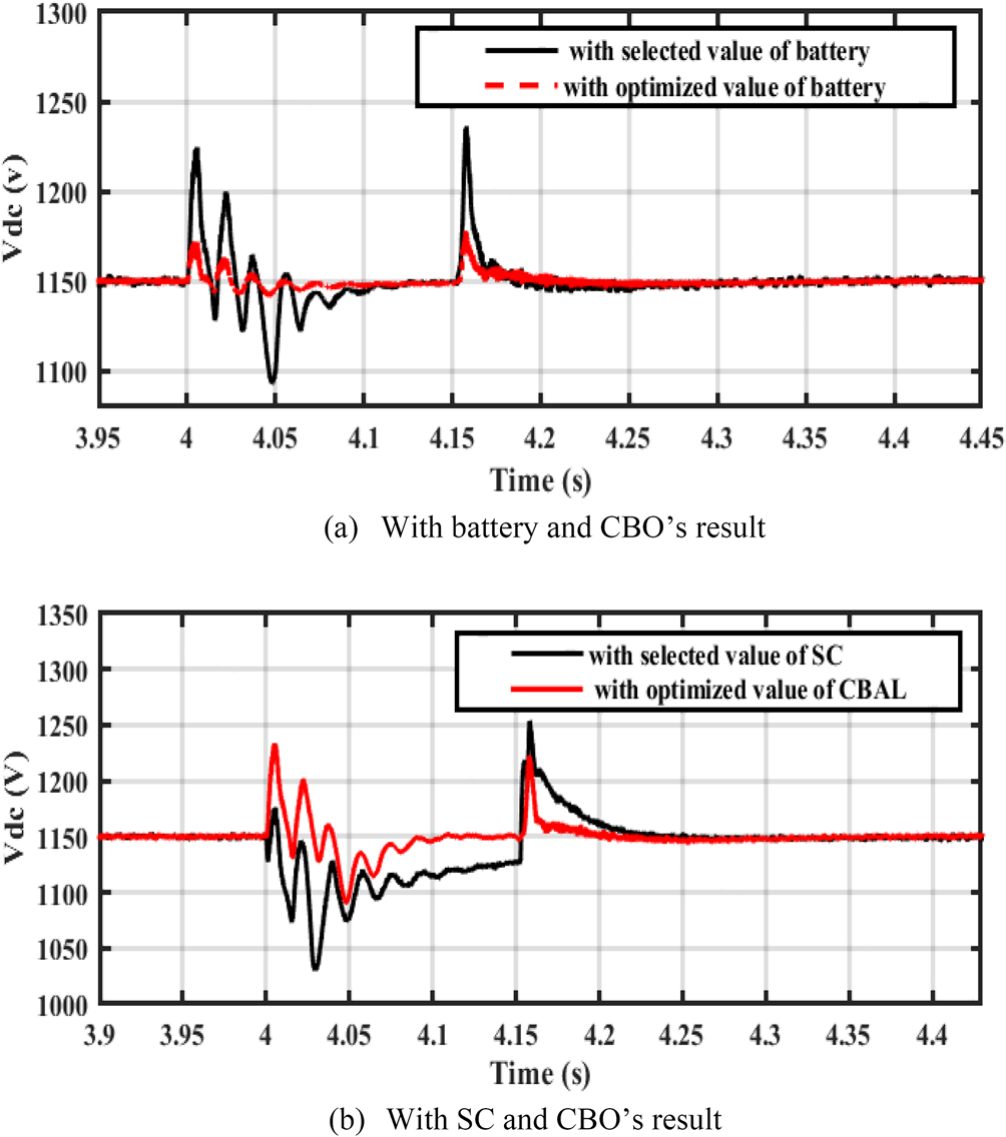
DC-voltage of WT system connected to the grid.

**Figure 21:**
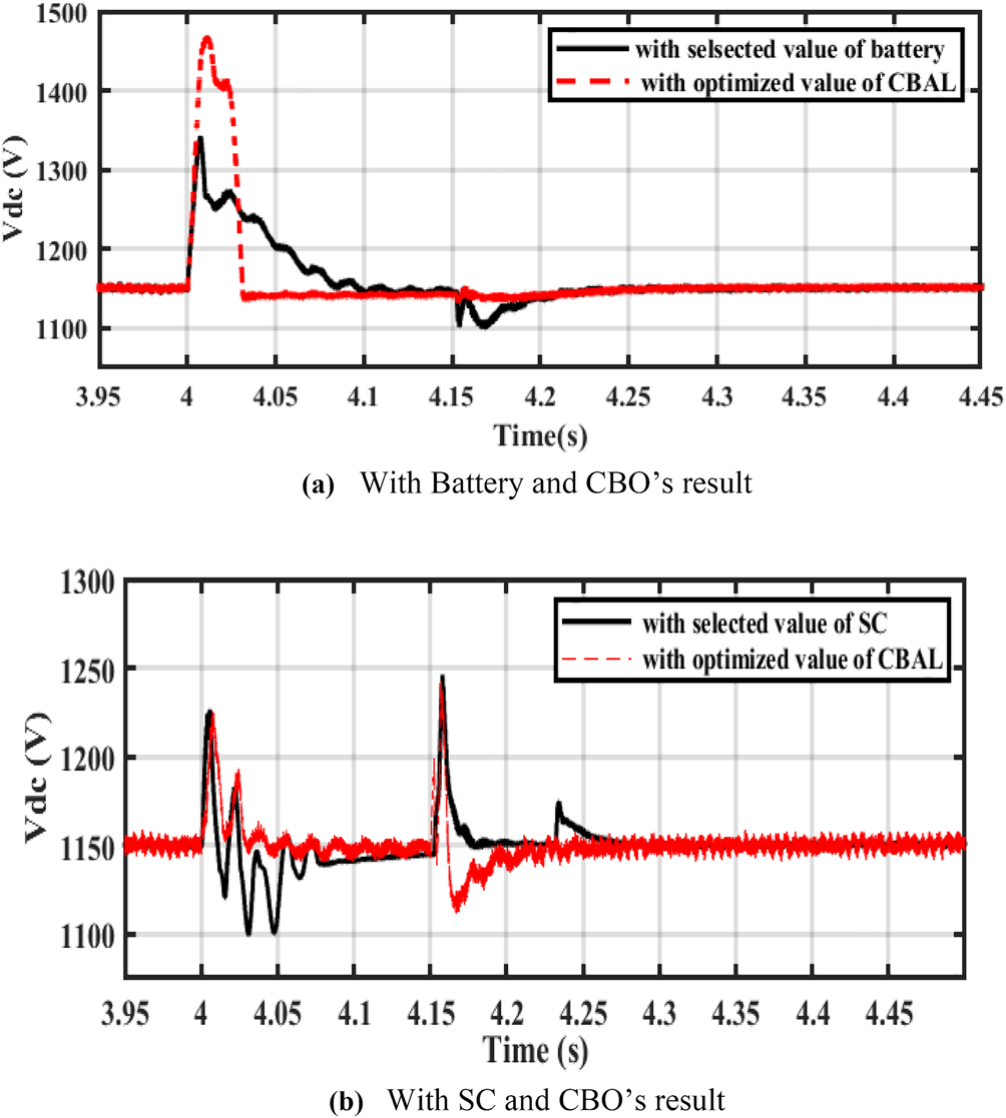
DC-voltage of PV/WT system connected to the grid.

It is noticed from the Table that the ripples in the DC bus voltage are more enhanced by the cropped values from CBO. Figure 19 shows ripples in the DC bus voltage of a PV system linked to the grid. Figure 19a illustrates that the ripples are improved by the CBO cropped rating of the battery. Figure 19b shows the same effect of the CBO’S cropped super capacitor rating on the voltage ripples. For WT systems connected to the grid, Fig. 20a,b demonstrate that the CBO’S cropped ratings of the battery and super capacitor more enhance the voltage ripples. Figure 21a,b shows that CBO’s cropped results improve voltage ripples in hybrid PV-WT systems connected to the grid.

## Conclusions

The article has studied the effects of three LVFRT schemes (CB, battery, and SC) to improve the performance of PV, DFIG-WT, and PV/WT on-grid systems during symmetrical and asymmetrical fault scenarios. According to simulation results, SC keeps the DC voltage, especially of the hybrid system at its rated value. The battery stores excess power in the DC bus of three systems to keep the DC-link voltage at a reference level. The CB circuit enhances LVFRT, but the excess power is consumed in the resistance. In its presence, SC keeps DC voltage at its rated level, decreases oscillations of active power, and the system reaches stability faster. At last, it can be confirmed that the Coot Bird Optimizer is an efficient tool to decide the optimal size of battery, supercapacitor, and the best gains of the bidirectional DC/DC converter PI-controller. By creating an experimental setup to evaluate the theoretical simulations and validate the acquired results by the CBO, it is aimed to further the current effort. Validation with HIL is necessary for understanding the complexities of real-time simulations. This aspect should be considered in the extension of this current endeavor, especially with the quite encouraging results from off-line simulations utilizing SIMULINK/MATLAB.

### Human and animal rights

This article does not contain any studies with animals performed by any of the authors.

### Supplementary Information

Supplementary Information.

## Data Availability

The data that support the findings of this study are available from the corresponding author upon reasonable request.

## List of symbols

${P}_{mec}$: Mechanical power of WT in (W)
$\rho$: Air density in (kg/${m}^{3}$)
R: Radius of the blade (m)
${v}_{w}$: Wind speed in (m/s)
$${c}_{p}$$: Power coefficient
β: Blade pitch angle in (deg)
$\lambda$: Tip speed ratio
$${V}_{dc}$$: DC voltage (V)
D: Duty cycle
$${C}_{PV}$$: Filter capacitor
${I}_{dc}$: Boost converter current (A)
$${I}_{L}$$: Inductor current (A0)
R, L: Internal resistance (Ω), and inductor (H)
${V}_{g}$: No-load battery terminal voltage (V)
p: Polarization voltage (V)
q: Battery capacity (Ah)
A, B: Exponential voltage exponential capacity constants.
${V}_{b}$,${i}_{b}$, ${R}_{b}$: Voltage, current, and internal resistor of the battery, respectively
${i}_{t}$: Take-out capacity
${v}_{dc}^{max}$: Maximum dc voltage
${\omega }_{s}$,${\omega }_{r}$: Angular speed, rotational speed of the rotor
${L}_{s}$,${L}_{r} {L}_{m}$: Stator, rotor, and mutual inductances, respectively
${L}_{Is} {,L}_{Ir}$: Stator leakage, and rotor leakage inductances
${n}_{p}$: Number of pairs
${I}_{P}$, ${I}_{D}$,: Shunt-leakage current (A), diode current (A)
${I}_{Ph}$, ${I}_{s}$: Light-generated current (A), saturation current (A)
q,${K}_{B}$: Electron charge (1.602 ${\times 10}^{-19}$), and Boltzmann’s constant(1.38 ${\times 10}^{-23} {\text{k}}$), respectively
${A}_{c}$: Ideality factor of the diode
T: PV cell temperature (${^\circ }_{C}$)
${I}_{PV}$, ${V}_{PV}$: PV cell current (A), and PV cell voltage (V), respectively
${i}_{ESD}^{*}$: ESD reference current
${V}_{dc}^{*}$: The reference value of dc voltage
${d}_{b}$: Signal used to turn on/off IGBT switches
$\Delta E$: Stored energy in a capacitor storage device
$\Delta t$: Period of changing energy
$$C$$: DC capacitor
${p}_{pcc}$: Active power delivered to PCC
${p}_{MP}$: Machine output active power
${V}_{max}$, ${V}_{Min}$: Maximum, and minimum dc voltage
$\Delta {v}_{dc}$: The difference in dc voltage
