# Supplementary material for: Consolidation of LVFRT capabilities of microgrids using energy storage devices
Source: Sci Rep. 2023 Dec 15;13:22294. doi: 10.1038/s41598-023-49659-0 (PMC10724191; doi:10.1038/s41598-023-49659-0)
Supplement: Supplementary file 1 — Supplementary Information. [file 41598_2023_49659_MOESM1_ESM.docx]

Appendix (A) parameters of PV and wind systems

| \| **Parameters of PV system** \| \| \| --- \| --- \| \| Nominal power (kw) \| 100 kw \| \| Cell number per module \| 96 \| \| Open circuit current \| Isc=5.96 A \| \| Open circuit voltage \| V_OC_ = 64.2V \| \| Maximum power current \| I_mp_= 5.58 A \| \| Maximum power voltage \| V_mp_=54.7 A \| \| Effective voltage of the grid \| V_g_=120 kV \| \| DC-link voltage \| V_dc_=500v \| \| DC-link capacitor \| C_dc_= 0.2130 F \| \| Grid frequency \| ɷ=2π*50 rad/s \| \| R filter of the inverter \| R=2 mΩ \| \| L filter of the inverter \| L= 250e-3 mH \| \| Inverter switching frequency \| f = 5 k HZ \| \| PI parameter of current loop \| K_p_=0.3, K_i_=20 \| \| voltage loop PI parameter \| K_p_=7, K_i_=800 \| | \| **Parameters of wind system** \| \| \| --- \| --- \| \| Nominal power (MW) \| 6 \| \| Nominal DC link voltage (V) \| 1150 \| \| Dc link capacitor (F) \| 10000*10^6^ \| \| dc link voltage controller k_p_ \| 8 \| \| dc link voltage controller, k_I_ \| 400 \| \| GSC current regulator, k_p_ \| 0.83 \| \| GSC current regulator, k_I_ \| 5 \| \| RSC current regulator, k_p_ \| 0.6 \| \| RSC current regulator, k_I_ \| 8 \| \| Var regulator, k_I_ \| 0.05 \| \| Voltage regulator gains k_I_ \| 20 \| \| Frequency of GSC (HZ) \| 2700 \| \| Frequency of RSC (HZ) \| 1620 \| |
| --- | --- | --- | --- | --- | --- | --- | --- | --- | --- | --- | --- | --- | --- | --- | --- | --- | --- | --- | --- | --- | --- | --- | --- | --- | --- | --- | --- | --- | --- | --- | --- | --- | --- | --- | --- | --- | --- | --- | --- | --- | --- | --- | --- | --- | --- | --- | --- | --- | --- | --- | --- | --- | --- | --- | --- | --- | --- | --- | --- | --- | --- |
